# Hotspots for mutations in the SARS-CoV-2 spike glycoprotein: a correspondence analysis

**DOI:** 10.1038/s41598-021-01655-y

**Published:** 2021-12-08

**Authors:** Mohammad Reza Rahbar, Abolfazl Jahangiri, Saeed Khalili, Mahboubeh Zarei, Kamran Mehrabani-Zeinabad, Bahman Khalesi, Navid Pourzardosht, Anahita Hessami, Navid Nezafat, Saman Sadraei, Manica Negahdaripour

**Affiliations:** 1 Pharmaceutical Sciences Research Center, Shiraz University of Medical Sciences, Shiraz, Iran; 2 Applied Microbiology Research Center, Systems Biology and Poisonings Institute, Baqiyatallah University of Medical Sciences, Tehran, Iran; 3 Department of Biology Sciences, Shahid Rajaee Teacher Training University, Tehran, Iran; 4 Department of Biostatistics, Faculty of Medicine, Shiraz University of Medical Sciences, Shiraz, Iran; 5 Department of Research and Production of Poultry Viral Vaccine, Razi Vaccine, and Serum Research Institute, Agricultural Research Education and Extension Organization (AREEO), Karaj, Iran; 6 Cellular and Molecular Research Center, Faculty of Medicine, Guilan University of Medical Sciences, Rasht, Iran; 7 Biochemistry Department, Guilan University of Medical Sciences, Rasht, Iran; 8 School of Pharmacy, Shiraz University of Medical Sciences, Shiraz, Iran; 9 Department of Pharmaceutical Biotechnology, School of Pharmacy, Shiraz University of Medical Sciences, P.O. Box 71345-1583, Shiraz, Iran

**Keywords:** Computational biology and bioinformatics, Evolution, Structural biology

## Abstract

Spike glycoprotein (Sgp) is liable for binding of the severe acute respiratory syndrome coronavirus 2 (SARS-CoV-2) to the host receptors. Since Sgp is the main target for vaccine and drug designing, elucidating its mutation pattern could help in this regard. This study is aimed at investigating the correspondence of specific residues to the Sgp_SARS-CoV-2_ functionality by explorative interpretation of sequence alignments. Centrality analysis of the Sgp dissects the importance of these residues in the interaction network of the RBD-ACE2 (receptor-binding domain) complex and furin cleavage site. Correspondence of RBD to threonine500 and asparagine501 and furin cleavage site to glutamine675, glutamine677, threonine678, and alanine684 was observed; all residues are exactly located at the interaction interfaces. The harmonious location of residues dictates the RBD binding property and the flexibility, hydrophobicity, and accessibility of the furin cleavage site. These species-specific residues can be assumed as real targets of evolution, while other substitutions tend to support them. Moreover, all these residues are parts of experimentally identified epitopes. Therefore, their substitution may affect vaccine efficacy. Higher rate of RBD maintenance than furin cleavage site was predicted. The accumulation of substitutions reinforces the probability of the multi-host circulation of the virus and emphasizes the enduring evolutionary events.

## Introduction

Severe acute respiratory syndrome coronavirus 2 (SARS-CoV-2) is the new member of beta coronaviruses^1^. It has emerged in Wuhan, China causing the ongoing outbreak of COVID-2019 (coronavirus disease of 2019)^2^.

Despite many suggestions and efforts such as social distancing^3^, existing-drug repurposing^2,4–6^, novel drug development^7–9^, and utilizing the plant-derived components^10^, the most promising way out of the pandemic seems to be vaccine development^11^. Vaccines should induce a long-lasting memory with minimal side effects. Such a vaccine candidate demands a careful selection of epitopes from the existing repertoire of the viral determinants^12^.

The most focal candidate for vaccine design is spike glycoprotein (Spg)^13^. Spike is a surface glycoprotein (~ 1300 amino acids) with vital roles in the pathogenicity of SARS-CoV-2. Receptor (angiotensin-converting enzyme 2; ACE2) binding, proteolytic activation of Sgp, and deliverance of the conserved fusion peptide into the host cell membranes are pre-internalization events mediated by the spike. At each step, the congregation of mechanisms and strategies are progressed^13–16^. Collectively, the virus entry into the host cells demands a splendid choreography of multifaceted pre-infection events^17^.

One of the main obstacles facing the vaccine design is antigenic drift^18,19^, which is highly pronounced in the RNA viruses due to their unstable genome^20^. In such situations, harnessing fast and reliable approaches that could predict emerging mutations are highly amenable. Several groups have attempted to distinguish the antigenic determinant of the Sgp. On the other hand, genomic data from all over the world evidenced a clonal and rapid in-human evolution of the SARS-CoV-2^21–23^. Various substitutions are continuously reported in the spike sequence^24^. This flexibility of the coronavirus genome warns about a great risk of infection severity and also foretells the possibility of vaccine^10,25,26^ or therapeutics^27^ failure.

Although mutations in any open reading frame of the virus genome could have implications on the severity or transmissibility of SARS-CoV-2, the insertions, deletions, and certain substitutions in the spike sequence could be of major concern. Examples of such substitutions include the dominant variant identified in the United Kingdom, known as B.1.1.7 (alpha variant). This variant holds mutation of N501Y; this mutation is also found in other variants of concern (VOCs)^28^ including South African 501Y.V2; B.1.351^29^ (beta variant), Brazilian 501Y.v3; P.1 (gamma variant)^30^. The variant is more transmissible and has been estimated to have a growth rate of 40 to 70%^31^. The N501Y governs an increasing receptor affinity^32^, which accents the eminence of special mutations at certain positions.

Overall, Sgp -similar to other proteins- is a critical combination of a complex web of ionic interactions, hydrophobic interactions, hydrogen bonds, and many other factors^33^. This protein’s holistic property is tightly entitled by its amino acid composition, which further dictates the secondary and tertiary structures and subsequently the function of the protein, which is subjected to natural selection. However, a selective constraint on a single site of a given protein can be interpreted in the context of its other building blocks. Since any substitution may affect the rest of the protein, the first changes may be affected again subsequent to the modifications, leading to a complicated web of reaction loops; which is indicative of a tangled bank of amino acid interactions. This issue introduced the phenomenon of evolutionary “stokes shift”; in which a protein as a whole entity, tends to make the resident amino acid(s) gradually stable^34,35^. Although the differences between emerging sequences and homologs are obvious and easy to spot through sequence comparisons, it would be appealing to define the corresponding residues and to inspect their substitutions. The corresponding residues make a target sequence odd and have key roles in the sequence function or are likely the main targets of evolution. We hypothesized that these sorts of substitutions are unique characteristics of proteins. Additionally, these residues may play an important role in the web of interactions in the protein; such substitutions might more effectively come into play in the way the Sgp_SARS-CoV-2_ behaves. This dramatically shapes the queries on how these amino acid substitutions are associated with the eccentric behavior of emerging sequences; more importantly, whether these substitutions are going to be stable or tend to be modified.

The corresponding residues can be singled out through sequence alignment by principal component analysis^36^. The aim of this study was investigating the correspondence of specific residues to the sequence of the Sgp_SARS-CoV-2_ by featuring the corresponding residues in the sets of aligned sequences. These data were complemented by the structural data to better grasp the importance of singled-out residues. The RBD and furin cleavage site were mainly focused here owing to their importance^37^. The study further discusses how these residual changes shape some critical traits of the Sgp_SARS-CoV-2_.

## Results

### Sequence data

Sgp_SARS-CoV-2_, a 1273 amino acid long sequence, is divided into five distinct domains as shown in Supplementary Table S1. The available SARS-CoV-2 Sgp homologous sequences were collected in the libraries of non-redundant sequences (proteins of similar length) based on the hidden Markov model profiling to cluster the complete sequences of spike proteins.

To better focus on domains of the protein, the sequences of the divided domains were searched against the databases separately. The search results were used to build the non-redundant libraries of sequences. Each library included sequences of similar length and e-value lower than 10^–4^. A preliminary review of the libraries showed that the libraries of RBD and N-terminal domain (NTD) were mostly occupied by beta coronaviruses, while other libraries contain more divergent members. Clustering experiments—in the following section—will better assess this issue.

The disparity index test showed a homogenous pattern of substitution for all datasets (data not shown); therefore, all sequences were retained for further evaluations and considered suitable for alignment approaches.

### Clustering the sequences

To define the relationship between sequences, each library was clustered based on the strength of their all-against-all pairwise sequence similarities. The network-based clustering approach also identified the closely related sequences and divided them into separate groups. Members of the sequence libraries in this section belong to coronaviruses excluding the SARS-CoV-2 (the limitation strategy of BLAST). The sequence collections are uniform in length and are the result of HMM profiling by querying the Sgp_SARS-CoV-2_.

**Figure 1:**
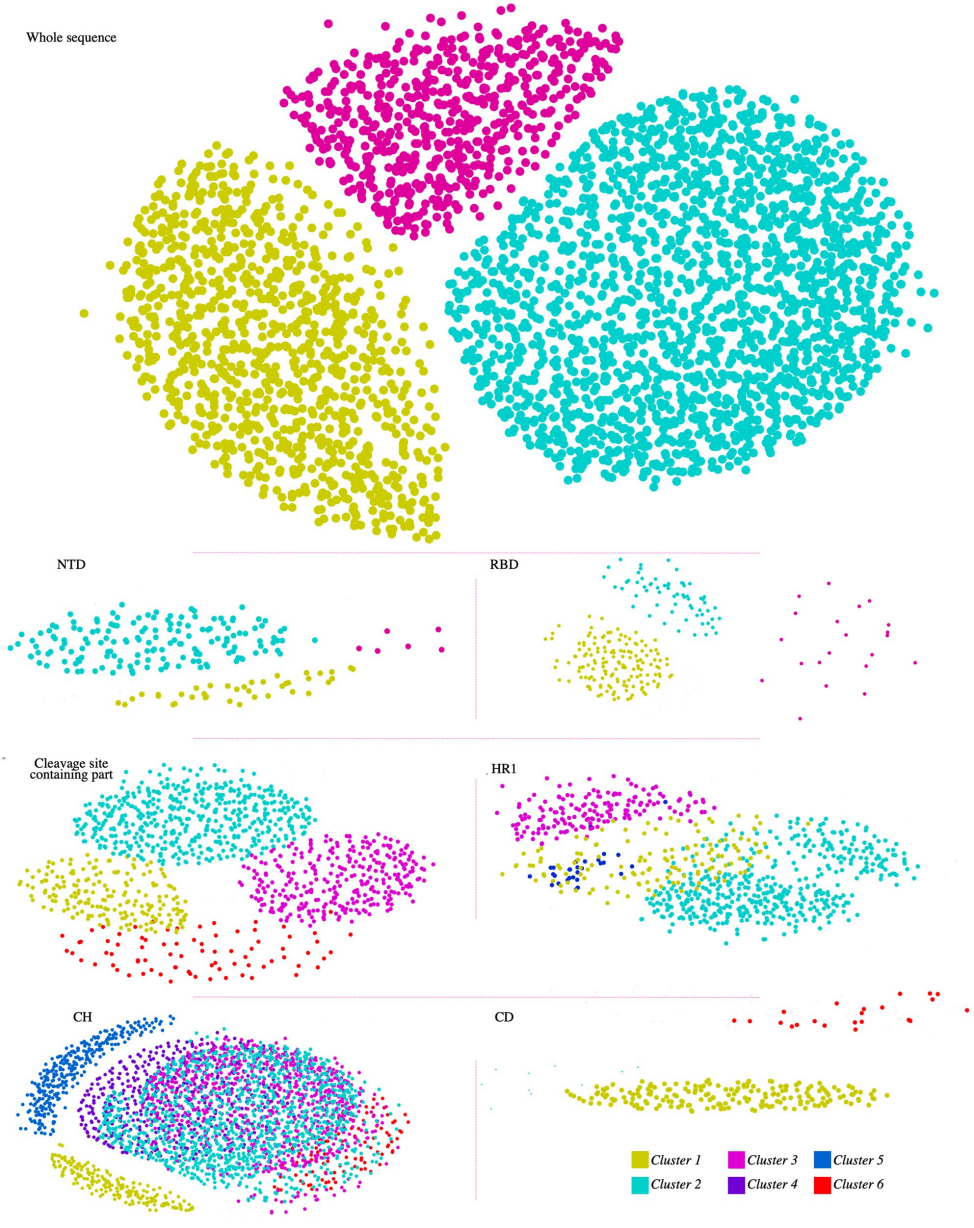
Visualization of the CLANS analysis results. Each panel shows the graphical two-dimensional representation of each dataset. Nodes represent the sequences in the analyzed dataset. Each sequence set includes the related domain and the corresponding homologous sequences derived from HMM profiling. The clusters are the results of the network-based clustering function of the CLANS software. The upper panel shows the clustering analysis from full-length sequences, and other panels are clusters from just the identified domain (labeled on the bottom left of each group). The nodes are colored based on the number of clusters (color key at bottom right). The sequence of Sgp_SARS-CoV-2_ or its domains is involved in cluster 1 in each group, except for the N-terminal domain (NTD); which is not involved in any cluster. The details of the clusters are summarized in Table 2.

Alpha, beta, gamma, delta (if existed), and unclassified (UC) sequences formed completely separate clusters (Fig. 1).

As illustrated in Fig. 1, when the dataset of the whole sequence of the Spgs was clustered, three groups were assigned. The results showed the true separation of beta-coronaviruses from other genera (cluster 1); as alphacoronaviruses were collected in cluster 2, and gamma-coronaviruses were collected in cluster 3. In contrast to the complete sequence, when some small segments of the protein were administered, the clustering approach yielded more specialized groups. The datasets derived from HMM profiling were clustered in more tangled sections when going through the C-terminal of the protein. The clustering results clearly showed that NTD and RBD segments are divided into distinct groups. The distinct groups are affiliated to beta-coronaviruses, reflecting the specificity of these domains even in one genus.

Interestingly, the NTD of SARS-CoV-2 does not involve in any identified cluster, reflecting the major disparity between NTD_SARS-COV-2_ and the other homologous sequences. Contrary to the N terminal, the C-terminal segments including CH, CR1, and CR2 involved virtually all groups of coronaviruses suggesting that these are the general determinants of spike (Fig. 1). Among all domains, the CH domain was the most scattered group. The details of the clusters, including the total number of sequences of each group, are summarized in Table 1; the total number of sequences and sequence IDs are provided in Supplementary Data 1.

**Table 1:** Details of clustered sequences.

Domain name	Cluster1	Cluster2	Cluster3	Cluster4	Cluster5	Cluster6	Total sequences
16–305 NTD	Beta (33), Alpha (2), UC (4)	Beta (157)	UC (6)	–	–	–	202
330–521 RBD	Beta (139), UC (10)	Beta (44), UC (17)	Beta (17), UC (3)	–	–	–	230
522–907	Beta (179), Alpha (2), UC (7)	Beta (455), Alpha (2) UC (23)	Beta (294), UC (8)	Alpha (74), Delta (1), UC (1)			1046
908–986 HR1	Beta (117), Alpha (1), UC (13)	Alpha (412), Delta (25), UC (16)	Gamma (164)	Gamma (29)	Alpha (22)		799
986–1035 CH	Beta(182), Alpha (2), UC (6)	Alpha (1114), Delta (72), UC (22)	Alpha (846), UC (6)	Beta (446), Alpha (2), UC (8)	Beta(300), UC(8)	Delta(89), UC(5)	3108
1076–1141 CD	Beta (180), Alpha (2), UC (6)	Beta (6), UC (4)					198
Whole sequence	Beta (900), Alpha (4), UC (26)	Alpha (1678), Delta (162), Gamma (4), UC (33)	Gamma (574)				3381

These results along with considering the sequence diversity within populations and subpopulations, suggest that the domains corresponding to the NTD show more diversity than the C-terminal (Table 2). Amongst, distinct patterns of diversity in RBD are noticeable.

**Table 2:** Distances and sequence diversities within different coronavirus populations.

Region	Group	Mean diversity in the subpopulations	Mean diversity in entire population^a^
16–305	Alpha	0.78	0.81
	Beta	0.93	
	Unclassified	0.8	
330–521	Beta	0.52	0.57
	Unclassified	0.84	
522–907	Alpha	0.82	0.82
	Beta	0.76	
	Unclassified	0.85	
908–985	Alpha	0.73	0.82
	Beta	0.67	
	Gamma	0.35	
	Delta	0.73	
	Unclassified	0.81	
986–1035	Alpha	0.18	0.29
	Beta	0.31	
	Gamma	0.28	
	Delta	0.19	
	Unclassified	0.34	
1076–1141	Beta	0.43	0.46
	Unclassified	0.63	

^a^The total number of analyzed sequences is mentioned in Table 1.

### Sequence alignments and correspondence analysis

A comparative analysis of the sequence libraries was conducted to find corresponding residues in each alignment set. Therefore, the minimal requirement was multiple sequence alignment, which was done for each library separately. The datasets were purged for duplicated sequences before the alignment process. The alignments were represented by sequence bundles as a visualization technique to view the one-to-one relationship between the sequences. This visualization technique in combination with correspondence analysis allows for saliently exploring physical properties and location of specific amino acids in respective positions.

To identify distant covariant sites in multiple sequence alignments (MSAs), a correspondence analysis was performed. This analysis provides a lower-dimensional representation of the alignment data in a scatterplot. The most striking observation that emerged from correspondence analysis was the dependencies of major domains (RBD, NTD, and furin cleavage motif) to a few residues (Table 3). The majority of the corresponding residues are structurally part of coils. Some residues occurred only once in our dataset, suggesting the existence of unique and specific mutations in the Sgp_SARS-CoV-2_ (total number of aligned sequences are mentioned in Table 3; the details of each sequence library on which alignments were built, is provided as Supplementary Data 2).

**Table 3:** Correspondence analysis. Introducing the key residues in each domain.

Domain (total number of nonredundant sequences)	Association	Secondary content	Occurrence	Sequences
16–305 NTD (85)	F32, T33	Coil	Once, 1.18%	A0A0U1WJY8 (*BtRs-BetaCoV/YN2013*), A0A1W5YKT9 (*Bat coronavirus,* UC)
330–521 RBD (230)	T500, N501	Coil, Coil	39 (45.88), 6 (7.06)	–
522–907 (1898)	NA			
660–700 (Furin cleavage motif: 194)	Q675, Q677, T678, A684	Coil, Coil, Coil, Extended strand	Once (0.52%)	–
908–985 HR1 (937)	NA			
986–1035 CH (344)	E990,	Helix	6 (1.74%)	A0A0P0INJ4 (*SARS-like coronavirus BatCoV/BB9904/BGR/2008*), D2DJW4 (*SARS coronavirus Rs_672/2006*), A0A0U1WHK9 (*BtRf-BetaCoV/HeN2013*), Q6R7Y6 (*SARS coronavirus NS-1*)
1076–1141 CD (34)	NA			

### RBD domain significantly corresponds to Thr500 and Asn501

**Figure 2:**
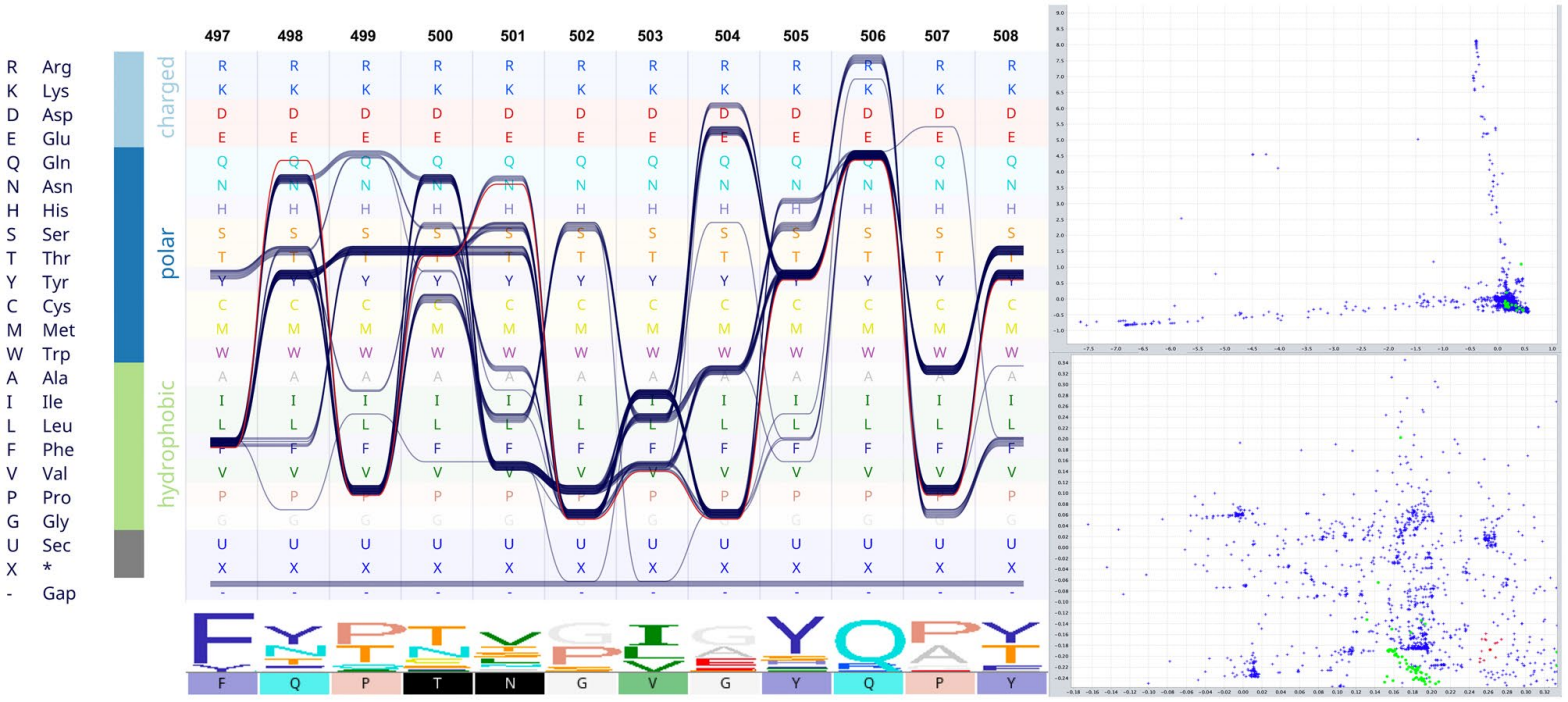
Combination of sequence bundle plot with sequence logo and correspondence scattered plot. The top left panel was initiated with different amino acids sorted by their chemical properties. Additionally, the top panel displays every sequence in the alignment as an individual continuous line, whose shapes correspond to the residues of that sequence. Multiple sequences that have the same residue are stacked on top of each other, thereby forming a thick bundle (conserved site). The sequence logo of the alignment section is also presented below the bundles and is followed by a color-coded sequence of Sgp_SARS-CoV-2_. The top left section shows the correspondence analysis scattered plot. Sequences are shown as green circles and sites as blue crosses. The Sgp_SARS-CoV-2_ sequence and nearby sites are selected (red colors). The selected sites (shown in red color) are mirrored in the sequence of Sgp_SARS-CoV-2_ at the lower panel (black boxes). Residues Thr500 and Asn501 are identified as significantly associated with Sgp_SARS-CoV-2_. The lower left is a zoom-in view of the selected site.

**Figure 3:**
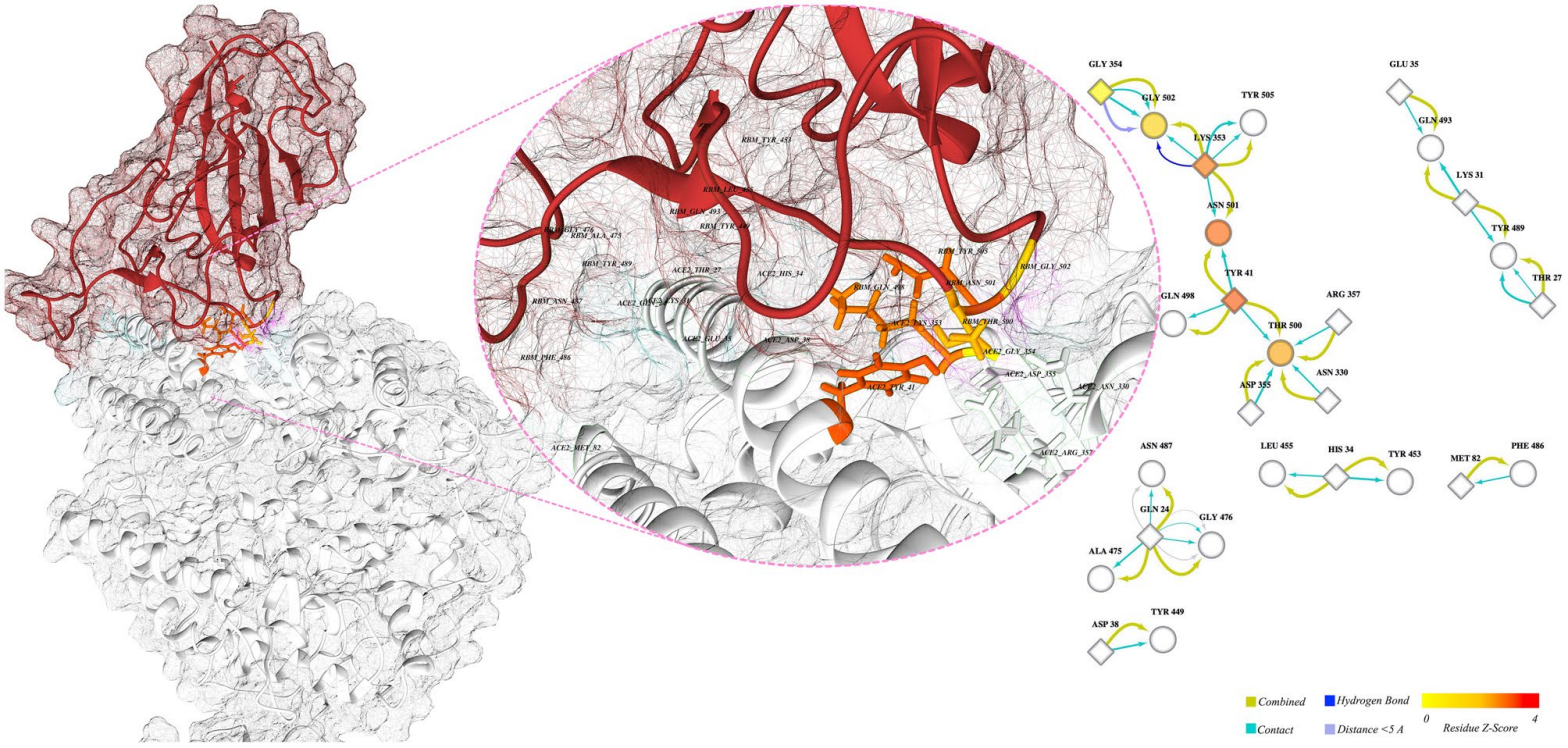
Cartoon representation of RBD in complex with ACE2 and interface residues. The left panel shows the cartoon representation of RBD (brownish ribbons) in complex with ACE2 (white ribbons) based on PDB entry 6VW1^38^. The middle image is a close zoom-in on the interface of the complex; residues involved in the interaction are labeled; surfaces are presented as transparent mesh. The right panel represents the interaction network of interface residues (the amino acids involved in the interaction between RBD and the receptor); ellipses and diamonds are related to RBD and ACE2, respectively. The node colors are based on Z-score (color key at the bottom left), white nodes have negative Z-scores. The colors of the ribbon in the structure are synchronized by the network. The significant Z scores are related to Tyr41 (2.76), Ans501 (2.532), Lys353 (2.26). The Z-score of Thr500, which is one of the corresponding sites, is 1.46.

A couple of corresponding sites were identified in the RBD domain viz. Thr500 and Asn501 and occurred in 45.9% and 7% of the MSA, respectively (Fig. 2). These two residues are directly involved in the interaction of RBD and ACE2 (Fig. 3). The interface residues in RBD and ACE2 complex are defined and labeled in Fig. 3. Moreover, when coupled with centrality evaluations, a significant Z-score endorsed on Asn501 (Z-Score: 3.008), reflecting a likely important role for this residue (Supplementary Table S2). The replacement of previously defined residues in these positions by Thr and Asn is a relatively radical substitution based on the Grantham distance matrix (Supplementary Table S3).

**Figure 4:**
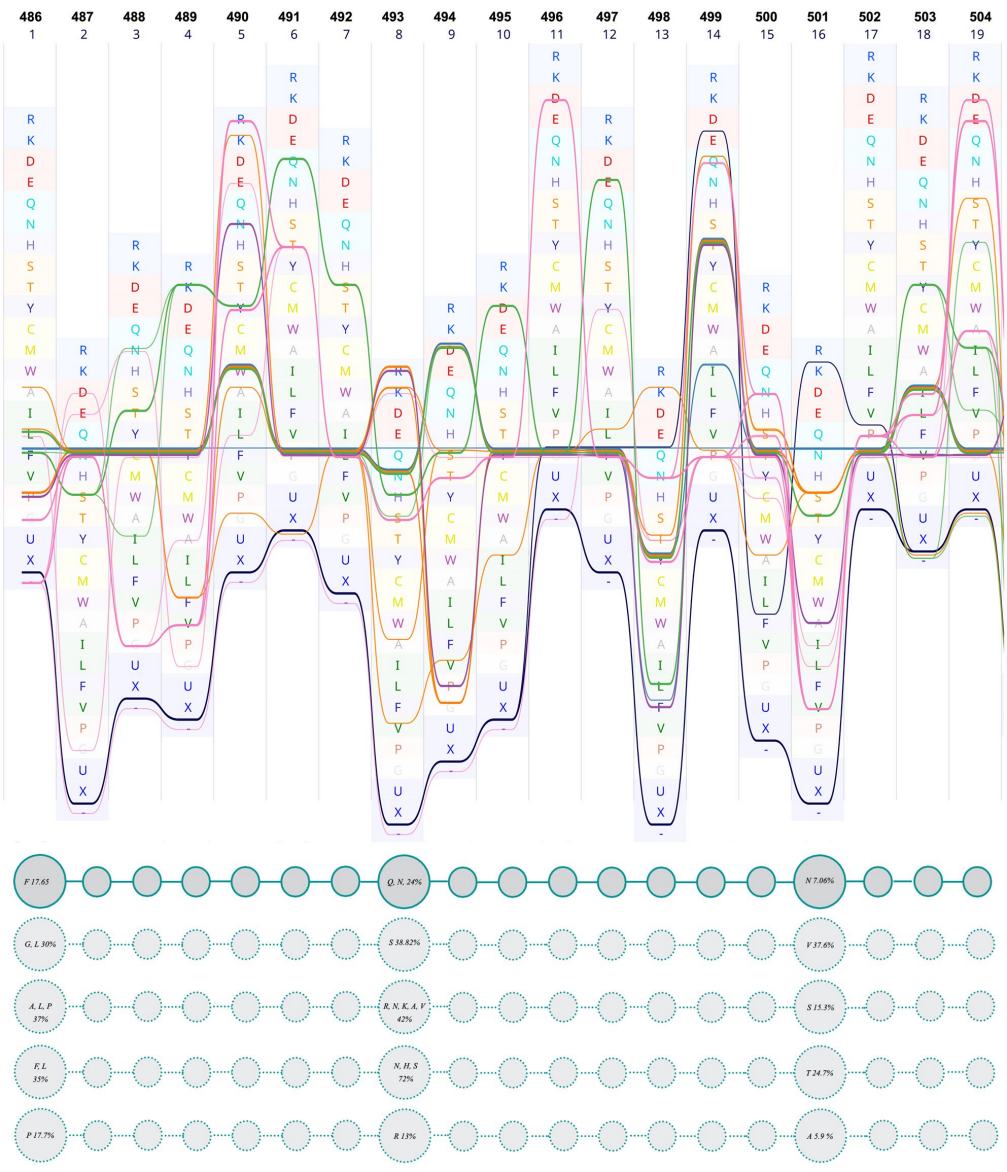
Multiple sequence alignment of RBM. In this figure, the sequence of RBM_SARS-CoV-2_ is set as the reference (upper panel). The lower panel schematically represents the respective residues and their location. The first line of circles presents the segment of RBM_SARS-CoV-2_, the other four lines represent four other groups of sequences at which those aforesaid positions are occupied by other amino acids. The numbers in the circles are the sum of the occurrence percentage of respective residues.

Herein, as well as two aforesaid positions, two other positions, namely 486 (Gln) and 493 (Phe), were evaluated, because they are all involved in receptor-ligand interaction. These are relatively variable sites (Fig. 4) and were introduced as major determinants for host range determination and tissue tropism in the earlier studies^38^. Sequence bundle visualization of MSAs allowed us to extrapolate the harmonious location of the residues in these sites.

The position of Asn501 in Sgp_SARS-CoV-2_ is mostly occupied by Thr in other sequences (for example 6ACG; SARS-CoV^39^). In our dataset, the sequences containing Asn at the same position include A0A023PTS3, A0A023PUW9, A0A2D1PXC0, U5WHZ7, and U5WLJ7. The striking observation is that all these sequences belong to the viruses that are hosted by *Rhinolophus affinis* (intermediate horseshoe bat).

We speculate that these three positions work in balanced harmony. To account for their relation and coordination, these positions were named from N-terminal to C-terminal as position 1 (F486 Sgp_SARS-CoV-2_), position 8 (493 Sgp_SARS-CoV-2_), and position 16 (501 Sgp_SARS-CoV-2_) (Fig. 4). As is evident in Fig. 4, position 1 is mostly occupied by polar amino acids, position 2 is always occupied by polar amino acids, and position 3 is always occupied by hydrophobic amino acids. These results show the existence of a striking harmony in the respective positions.

The identified residues are parts of experimentally defined epitopes^40–43^ (Supplementary Tables S5, S6).

### Conservancy rate of receptor-binding motif (RBM) and the remaining section of RBD

The existence of corresponding residues in critical positions mentioned in the preceding paragraphs has prompted us to answer a critical question: why these substitutions were singled out through the correspondence analysis. We attempted to examine the variation in the evolutionary rate of different sites of RBD.

This section sketches the physicochemical properties of the RBD, derived from sequence data to estimate the evolutionary rate. The data presented here is derived from the sequence data; structural data were also included (for comparison) (Supplementary Data 3).

To explore the differences between ten variables of surface accessibility, flexibility, buried area, as well as CX, DPX, CN, Bfactor, accessible surface area, similarity scores, and identity scores, the non-parametric Mann–Whitney U test was performed for each variable. The purpose of this assessment was to investigate whether the variables are significantly different between subpopulations of receptor-binding motif (RBM) and the remaining part of RBD. The results showed that the distribution of flexibility, ASA, CX, Average Bfactor, identity scores, and similarity scores were not the same in the two populations (details are provided in Supplementary Data 3). Additionally, the test was performed for comparing the values of three focused residues of RBM [(F486 Sgp_SARS-CoV-2_), (493 Sgp_SARS-CoV-2_), and (501 Sgp_SARS-CoV-2_)] with the remaining residues. Additionally, the Mann–Whitney U test was used to investigate the differences between those three focused residues of RBM [(F486 Sgp_SARS-CoV-2_), (493 Sgp_SARS-CoV-2_), and (501 Sgp_SARS-CoV-2_)] and the remaining parts of RBM or the remaining parts of RBD. The results suggest that the distribution of all ten parameters was the same in RBM. Further, for the three focused residues, only similarity and identity scores differed significantly (p-value 0.05, Supplementary Data 3). No differences were observed between the three focused residues and the remaining parts of RBM.

**Figure 5:**
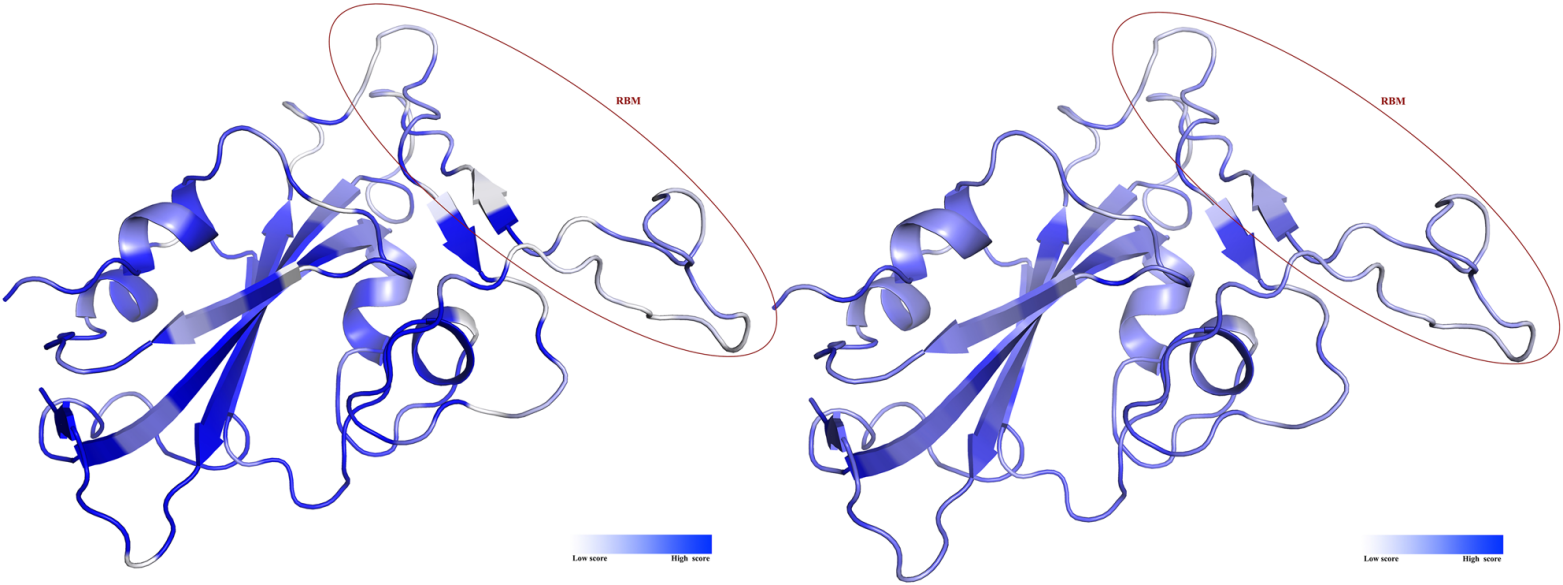
Stereo view of RBD. The ribbon on the left panel is colored based on the identity score; and the ribbon on the right panel is colored based on the similarity scores. The scores were calculated based on BLOSUM62 by ProtSkin.

Moreover, Fig. 5 shows that RBM is composed of similar amino acids in the dataset of aligned sequences (Fig. 5, right panel), and identical residues are rare in this motif.

Collectively, data in this section revealed the existence of evolutionary rate variations among RBM in comparison with the whole RBD.

### The furin binding motif is modified in favor of furin activity

**Figure 6:**
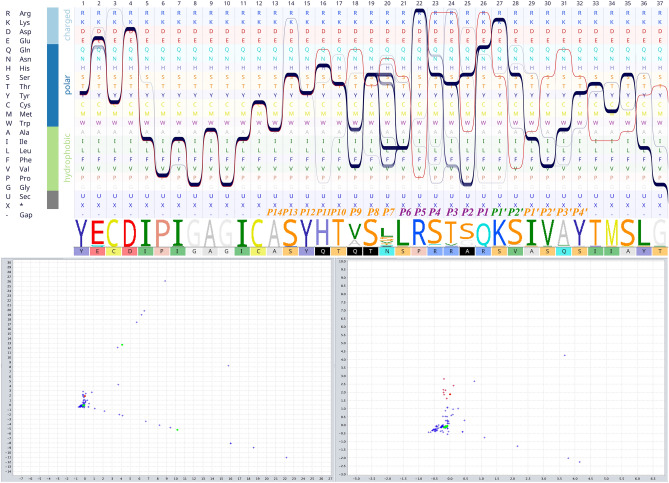
Bundle representation of furin cleavage site and correspondence scatter plot. The left panel shows the one-to-one comparison of sequence MSA of the furin cleavage sites of different coronaviruses. The 20 amino acids of the furin cleavage motif are depicted by different colors on the top of the sequence alignment logo (P stands for the position). The color scheme of fonts is based on reference^38^. Amino acids are sorted based on their biochemical properties. Each continuous line represents a sequence in the alignment. The red line is the sequence of the Sgp_SARS-CoV-2_ furin cleavage site. The lower panel shows the correspondence scatterplot and a close look at the selected correspondence sites (left and right panels, respectively). The corresponding residues are in black boxes in the sequence of Sgp_SARS-CoV-2_ on the left panel. X and Y axes are the first and second principal components, respectively.

The sequence alignment section of the furin binding pocket of Spgs suggests a high level of the conservancy of this motif among the dataset. Surprisingly, the exception was the sequence of Sgp_SARS-CoV-2_ (red line in the alignment bundle in Fig. 6). In the evaluation of the furin cleavage site, the pattern introduced by the seminal work of Tian et al. and similar nomenclature was followed, because we found it plausible for explaining the properties of the furin binding motif of Sgp_SARS-CoV-2_. The authors explained the furin binding motif as a core region surrounded by two flanking boxes (see Refs.^44,45^). The core region is occupied by positively charged residues, and the flanking regions are more flexible and surface-accessible residues. The furin binding site significantly corresponds to Gln675, Gln677, Thr678, and Ala684 (Fig. 6), while these positions in the alignment are occupied by other amino acids. In the other words, these residues have occurred only once in the MSA. Therefore, it can be concluded that these non-conserved residues are likely species-specific. Notably, based on the Grantham replacement matrix, these substitutions are relatively conservative (Supplementary Table S3). These residues are also involved in some experimentally validated epitopes (Supplementary Tables S5, S6).

**Figure 7:**
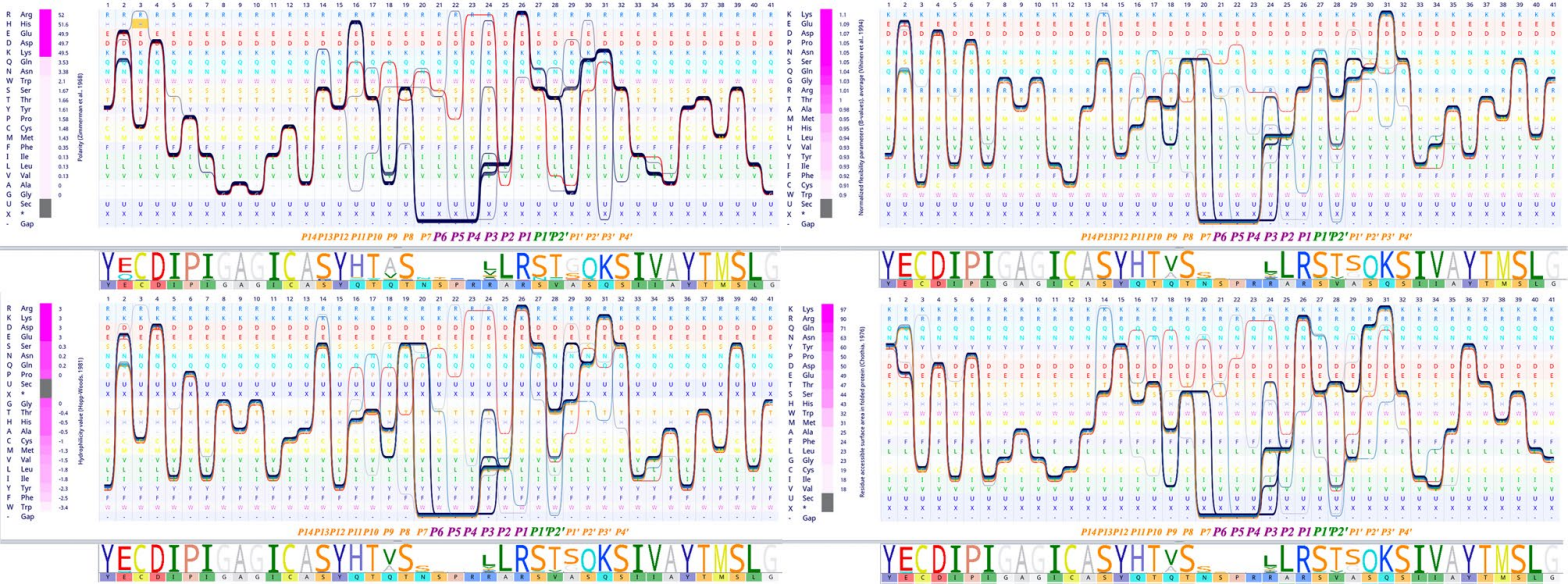
Comparison of the chemical properties of the furin cleavage site of SARS-CoV-2 spike glycoprotein with other coronaviruses. Four chemical properties of the furin cleavage site are compared with other coronaviruses by bundle illustrating the MSA. In each panel, the amino acids are sorted based on different chemical properties. The sequence logo and the sequence of SARS-CoV-2 spike glycoprotein furin cleavage sites are presented below each panel. The 20 amino acids of the furin cleavage motif are depicted by different colors on the top of the sequence alignment logo. The color scheme of fonts is based on the reference^38^. The red line in each panel is the sequence of the SARS-CoV-2 spike glycoprotein furin cleavage site.

The modification of the aforesaid residues resulted in vast modifications in the biochemical properties of the furin binding site (Fig. 7) of Sgp_SARS-CoV-2_. Figure 7 shows how corresponding residues, which are in positions 2, 8, 9, and 11 of the cleavage motif, alter the physicochemical properties of the furin binding motif. The core domain of the furin cleavage site is more positively charged and occupied by residues with high isoelectric points. The P1′, which is the exact cleavage site, is resided by alanine, a small hydrophobic amino acid.

As illustrated in Fig. 7, polarity, flexibility, hydrophilicity, and surface accessibility of the flanking regions were increased upon mutations. It is evident in Figs. 6 and 7 that the corresponding residues are, in part, responsible for these modifications.

In comparison with other coronaviruses, the furin binding site is more charged, more flexible, more hydrophilic, and more accessible.

**Figure 8:**
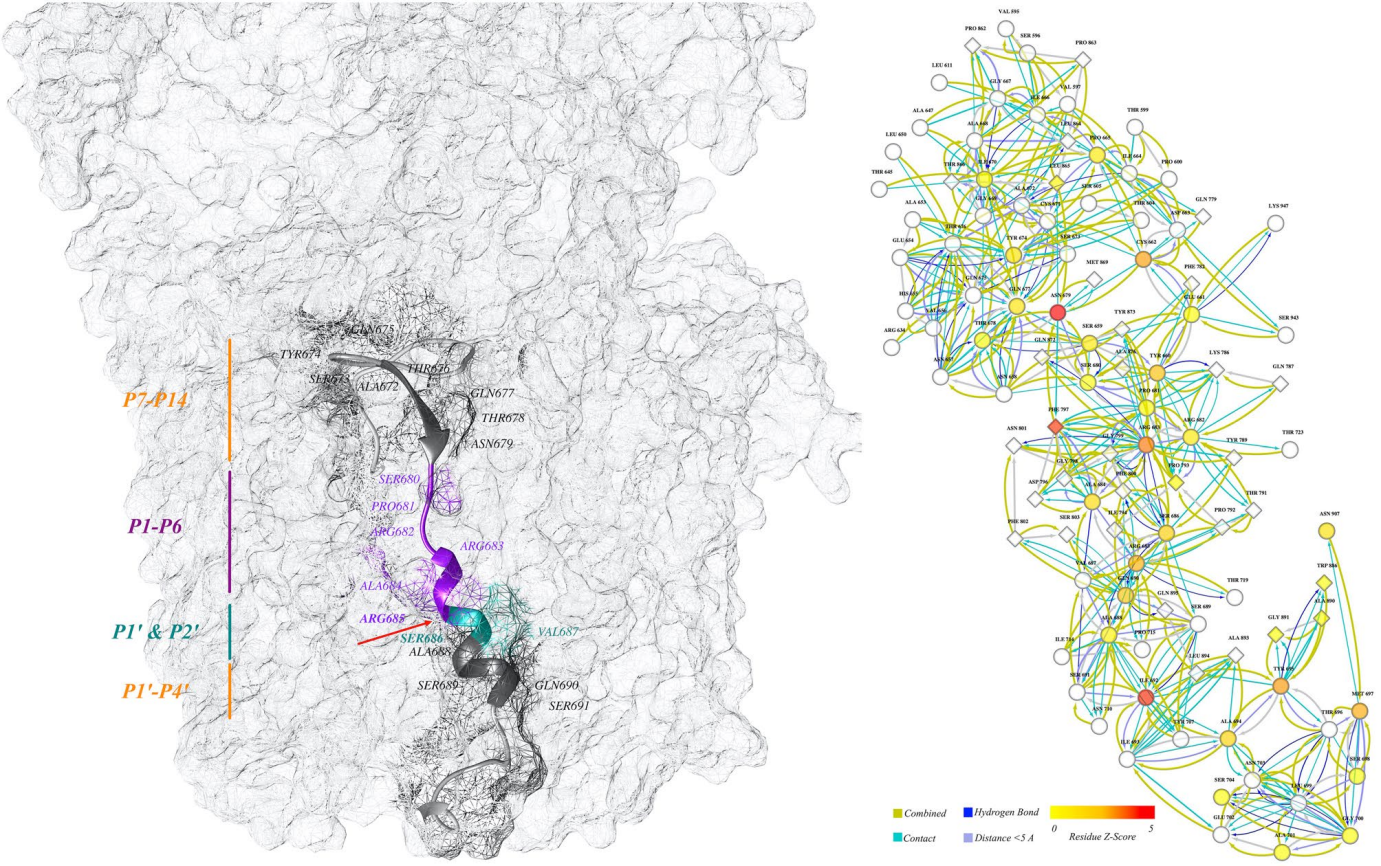
Cartoon presentation of the stalk of the SARS-CoV-2 spike glycoprotein. The left panel shows the stalk of the SARS-CoV-2 spike glycoprotein. The furin cleavage motif is discriminated against by colored ribbons; other ribbons were hidden for better presentation. The positions of the different boxes of the furin cleavage motif are illustrated by vertical lines; the color scheme is based on reference^38^. The different parts of the furin binding site are assigned by different colors of ribbons, and the red arrow shows the exact cleavage site between residues of Arg685 and Ser686. The right panel shows the network interaction of the furin binding site and other residues in the trimeric structure. The ellipses are residues of the binding site and diamonds are the residues on the nearby chain. The filled color is based on Z-score, and edges are different interactions (color key at the bottom). The cartoon representation of the protein is generated by Chimera (ver. 1.13); the network in the left panel is made by Cytoscape (ver. 3.7.2).

Besides, the interaction of furin binding motif with each other and with other residues generates a relatively complicated network (Fig. 8, right panel); central residues and their corresponding Z-scores are presented in (Supplementary Table S4). The Z-scores were calculated based on the free molecule. As shown in Supplementary Table S4, none of the corresponding residues from alignment analysis achieved a significant Z-score.

The substrate (furin binding pocket of Sgp_SARS-CoV-2_) and the proprotein convertase (furin) were docked. The resulted complex was used for centrality analysis. This complementary approach examined whether or not the corresponding residues attain significant centrality Z-score in the complex form. The results (Supplementary Table S4) showed that the Z-scores of centrality are significantly different in the two states (these are furin binding motif, free and in complex with furin).

### Tracking the substitutions in Sgp_SARS-CoV-2_

True SARS-CoV-2 sequences were collected by filtering the BLAST result of the RBD nucleotide sequence against all the available SARS-CoV-2 sequences to match records with expected values between 0 and 6e−26 and a query coverage between 80 and 100. The best model for describing this dataset was defined as Tamura three parameters. The probability of rejecting the dN = dS in favor of dN > dS or dN < dS was not significant, therefore no sign of positive or purifying selection was observed in sequence variants of Sgp_SARS-CoV-2_.

Tracking the substitution frequency of RBD, RBM, and furin cleavage site, after one year of in-host evolution, suggests no significant differences between these domains and other parts of the sequence (all defined substitutions are provided as supplementary data 5; the data was obtained from GISAID database).

## Discussion

Amino acid changes can range from biochemically similar (conservative substitutions), to dramatically dissimilar amino acids (radical substitutions)^10,46–48^. Among many substitutions that happen in a protein, few mutations are critical and are actual targets of evolution. A simple and fast method to find these sorts of substitutions might be helpful to understand the nature of emerging diseases, developing novel vaccines, and explaining the behavior of progressive virulence factors. In emerging viruses, the amino acid changes would drastically affect the sensitivity of protein to certain neutralizing antibodies or cause a vaccine or therapeutic failure^49^. Understanding the rate and positions of the mutations and even predicting the stability of certain parts of the protein is important for vaccine design, planning any therapeutic approach, and studying the nature of the emerging sequence. Furthermore, it is critical to define how these substitutions shape the novel traits of the emerging pathogen. Among pathogens, the genome adaptability of RNA viruses makes them more susceptible to jump to new hosts^50^. The spillover and transmissibility in these cases usually depend on the existence of virus receptor(s) on the host cells^51^; the more the receptor is conserved among different target species, the higher the virus is anticipated to spread. The recent examples within the last two decades are members of coronaviruses causing the SARS and the Middle East Respiratory Syndrome (MERS), and more recently SARS-CoV-2^14,52^.

The interactions of residues in a protein and protein–protein interface rule the maintenance of new substitutions, highlighting the existence of great harmony in the whole protein. In a MSA, the single sites include relatively less information than the entire MSA. Therefore, large and diverse datasets are required for detecting critical sites^53^. This paper appraised the alignment method along with principal component analyses (correspondence analysis) to describe the dependencies of small segments of Sgp_SARS-CoV-2_ to specific residues. The work also attempted to predict whether these substitutions would be stable or tend to be modified.

The multi-domain Spg is likely the most important determinant of coronaviruses, because it is responsible for the multi-step process of host recognition and tissue tropism^54^. This prevailing role has propelled the Spg to the forefront of the coronavirus infection investigations, vaccine design, and arrangement of therapeutic plans. While small modifications on RBD may significantly alter the host selection theme of the virus^36,55^, it has dramatically shaped queries on these little differences. Moreover, maintenance or changeable prophecy of the protein segments would allow us to select stable and effective epitopes for vaccine design or drug targets^56^.

Two adjacent Thr500 and Asn501 amino acids are the corresponding sites of RBD. Asn501 contains a significant Z-score of centrality, when the molecule is in complex with its ligand (whereas a free molecule does not obtain a significant Z-score), emphasizing the important role of this residue and context dependencies. These two amino acids are in the C-terminal proximity of RBM and are involved in the receptor-binding interface. Ascribing a central role for Asn501 corroborates the Li et al.^36^ argument in which the authors attributed the important role of human–human transition or host range determination to this residue. The authors especially highlighted the role of the side chain^36^. Additionally, significant differences in the Z-scores of centrality between free RBD and RBD-ACE2 complex confirmed the importance of this residue and the context wherein this site is involved. The more obvious evidence involves the alpha variant of SARS-CoV-2, in which the substitution of Asn501 with Tyr501 made the virus dominant^14^.

RBM is the receptor-binding region, isolated from the edge of RBD, with specific sites entangled in ACE2. Presenting the alignments as sequence bundles, which is a sequence-oriented technique, unveiled some hidden properties of the sequences. Our primary assumption for interpreting the conservancy features of RBD and especially RBM was that the less conserved residues would be more effectively involved in the host range determination of the virus, because conserved residues are present in all strains and may not centrally affect the jumping or the specific nature of the viral infection. Together with the role of mutation at a specific site, the effect of the amino acid coalition should be considered when discussing the protein properties. Fourteen positions in RBM are the key residues for binding of SARS-CoV to human ACE2^14^. In Sgp_SARS-CoV-2_, six out of fourteen residues are semiconservative compared to SARS-CoV: N439_SARS-CoV-2_ (R426_SARS-CoV_), L455_SARS-CoV-2_ (Y442_SARS-CoV_), F486_SARS-CoV-2_ (L472_SARS-CoV_), Q493_SARS-CoV-2_ (N479_SARS-CoV_), Q498_SARS-CoV-2_ (Y484_SARS-CoV_), and N501_SARS-CoV-2_ (T487_SARS-CoV_)^57^. The sequence data herein suggests that the RBD (and also NTD) is species-specific. Moreover, it appears that the presence of semi-conservative residues, which are the differed parts among previous beta coronaviruses, could have important roles and most likely has an influence on host range determination, tissue tropism, and the current rapid SARS-CoV-2 transmission in humans. Our focus on three hotspots of RBM, namely F486_SARS-CoV-2_, Q493_SARS-CoV-2_, and N501_SARS-CoV-2_, surmises the overall conserved physicochemical properties of RBM, caused by the collaboration of specific residues, leading to a successful viral attachment and cell entry.

The viral entry into susceptible host cells is a complex process^51^ and demands maintaining harmony between certain residues of the Spg; it is an indication of a complex tangled bank of amino acid interactions. The full functionality of a protein requires maintaining a balance between the physicochemical properties of major amino acids.

Viruses extracted from the sporadic SARS cases, during 2003–2004, all had asparagine at position 479 and serine at position 487; each virus was an independent cross-species event without the human-to-human transmission^36,58^. Based on these observations, Li et al.^36^ concluded that the side chain of the residue at 487 is a key factor for shaping severity (and likely human-to-human transmission)^59^. These positions in Sgp_SARS-CoV-2_ are replaced by Q493 and N501, respectively. The coexistence of amino acids with specific physicochemical properties could be a marker of the harmonious interaction of residues in this specific region. Therefore, the binding properties of RBM could be more complicated than has been thought earlier.

Similarity and identity scoring strategies reveal the existence of many substitutions in RBD (mostly conservative). The corresponding residues of RBD were found as parts of RBM in the alignment set. Evolutionary variation among different sites depends on various physicochemical properties of the amino acids including surface accessibility^60,61^, packing density, and flexibility^29,62–64^. Surprisingly, regarding the increased levels of surface accessibility and flexibility in association with a decreased level of contact density of RBM in comparison with the remaining parts of RBD, it can be concluded that RBM has a greater evolutionary rate. Therefore, it is evident that the harmonious interaction of residues goes far beyond a small motif. While the evolutionary rate of RBM is higher than the remaining part of RBD, it can be finally concluded that the residues in RBM are targeted by evolution, and other parts tend to preserve these substitutions.

Most VOCs are carrying substitutions in the 501 positions. For example, an emerging UK variant: B.1.1.7 harbors an N501Y mutation which increases the interaction of spike with ACE2 receptor^27,65,66^. The modifications along with increase the affinity of Sgp_SARS-CoV-2_ to the ACE2 receptor, cause failure of S gene targeting by molecular diagnostics; an example includes Thermo Fisher TaqPath COVID-19 assay^15^.

It is worth mentioning that this position is focused in our study and was defined by correspondence analysis including previous coronavirus sequences, which strongly highlights the usefulness and efficacy of our method.

Not only the amino acids of a protein but also hosts and viruses are in a tangled bank of interactions^67^. The successful completion of viral life spam highly depends on the host elements. In the case of coronaviruses and SARS-CoV-2, the cleavage of the spike by host proteases is important in the infectivity and host range modulation^68^. For instance, a study on MERS-CoV strengthened the concept that along with the virus receptor, the repertoire of proteases expressed by a given cell type, could significantly affect the infectivity^69^. Activation of the spike is a crucial step of the infection and depends on the host’s furin activity^70^. For example, despite the ability of MERS-CoV-related bat coronavirus, HKU4, to recognize the human receptor-dipeptidyl peptidase 4, the activation of this virus does not happen in humans, since the process demands additional exogenous trypsin^15^. Furthermore, the presence of glycan near the S1/S2 boundary may completely abolish the proteolytic priming of the virus^71^. Cleavage at different sites can occur in a different lifestyle of the virus during biosynthesis or virus entry; whenever it happens, it can critically affect the cell and tissue tropism as well as host range determination^14,39^. Sgp_SARS-CoV-2_ harbors a furin cleavage site at the S1/S2 boundary, which is treated during biosynthesis^14^.

Furin cleavage site is known as a consensus pattern of R-X-[K/R]-R⇓ (where X is any amino acid). However, all furin cleavage sites do not follow this pattern^38^. Exploring the first release of Sgp_SARS-CoV-2_ sequence data, at the first stages of the COVID-19 pandemic, evidenced a four residue insertion at the S1 and S2 boundary in comparison with other SARS coronaviruses^39^. Indeed, we examined this region as a broader motif of 20 amino acids.

An evolutionary conserved 20 amino acid motif could better describe the furin cleavage site as explained by Tian et al.^38^. Their seminal work also mentioned the conservancy of the physical property of this motif among mammals, bacteria, and viruses^14^. The motif was defined as a core region (P6–P2′) that fits into the catalytic pocket of furin and two flanking flexible solvent-accessible regions (P7–P14 and P3′–P6′). The core region determines the binding strength of the enzyme and its substrate; while the flanking regions provide the core region accessibility to furin. The alteration of residues in this motif would drastically affect the efficiency of furin cleavage^39^. It also may affect viral expansion, cell and tissue tropism, transmissibility, and pathogenicity^72^.

In the furin cleavage pocket, the balance maintenance between hydrophobicity and hydrophilicity is a fascinating characteristic of viral fusion proteins^47^. Our data showed that all modified properties are in favor of furin cleavage activity. It is worth mentioning that these differences are derived from exchanging the conserved residues (mostly radical substitutions) in the Sgp_SARS-CoV-2_ sequence along with the insertion of a short peptide. Similar to RBD, it could be hypothesized that these radical substitutions are targets of evolution, and other sets of substitutions are present for retaining these sites.

Radical substitutions are more probable to be chosen against conservative substitutions^73^. Additionally, organisms with a small effective population size tend to accumulate more radical substitutions than those with larger effective population size and more efficient natural selection^74^. Although the currently available database did not provide adequate information to trace a positive or a negative selection, it is not possible to predict the fate of the spike protein. Nevertheless, regarding the huge effective population size of the virus, the accumulation of conservative substitutions is expectable. Therefore, the modification of the furin cleavage site is more likely to happen, and the maintenance of RBD in the current composition is presumable. Moreover, different sites of the protein may face diverse environmental contexts, thus might have a dissimilar evolutionary fate. This assumption is consistent with our findings on the sequence diversity of Sgp_SARS-CoV-2_. Since the C-terminal of Sgp_SARS-CoV-2_ is located in a relatively constant microenvironment (viral envelope), a low diversity level can be observed in these segments.

Due to the proof-reading properties of RNA polymerases of coronaviruses^10,75^, the mutations are reduced in this family including SARS-CoV-2, relative to other RNA viruses. However, as many research groups are continuously monitoring the genomic diversity of SARS-CoV-2^10^, many mutations are indeed reported. The emergence of mutations in the Spg as the most antigenic determinant of coronaviruses, would cause antigenic drift and subsequently vaccine or drug stagnation. Previous research has demonstrated the altered capacity of some neutralizing antibodies against Sgp_SARS-CoV-2_ due to the recent mutations. None of the discussed residues in the present study was included in the set of evaluated mutations^76^. Furthermore, surveying the genomic database (https://www.gisaid.org/epiflu-applications/phylodynamics/) of SARS-CoV-2 revealed that more mutations are accumulated in the furin cleavage site (and its vicinity) than RBD, which confirms our assumption of maintenance and modification probability of RBD and furin cleavage site, respectively.

This paper shows how sequence-based computational approaches could be applied solely to extrapolate important features of an emerging sequence prior to availability of more complex costly structural information. The most prominent feature of this study is the data presentation, especially the visualization of the MSAs as sequence bundles. Dissemination of sequence data and coupling these observations with structural information manifests the usefulness of in silico tools to delve into important features of emerging virulence agents. Moreover, in silico tools hold great potential of screening bioactive components^77^ for inhibiting critical enzymes of the virus^78^ or other non-structural components through molecular docking or molecular dynamic simulations^79,80^.

A wide research ground is provided here for future studies to describe the dynamic and energetic features of sequence modifications and manifest the role of other nearby residues and their implications in the protein architecture as a whole. In this regard, the accumulation of various substitutions that occurred in Sgp_SARS-CoV-2_ could be a signature of long-lasting evolution. Given these enduring events, it could be hypothesized that the coronavirus has been confronted with different environmental contexts and thus, faced different evolutionary pressures. It is plausible for further studies to be focused on this assumption.

## Conclusion

The slight differences of SARS-CoV-2 with its close relatives, shape its distinguished characteristics that are responsible for the easy spread of the virus and its spillover. Within many residue substitutions, a few belonging to RBM and furin cleavage motif, were shown to be correlated with the corresponding domains. Our results implicated that singled-out residues may be the real targets of evolution and other substitutions tend to maintain these resident amino acids at certain positions. Residues in the consortium are responsible for explicit features of RBD and furin cleavage motif. The location of amino acids in certain positions revealed a tangled bank of amino acid interaction web. The compensatory role of amino acids may explain this harmonic localization. While the identified residues are parts of experimentally identified epitopes, it should be pinpointed that antibodies or vaccines that target the mentioned residues would remain effective.

While the initial molecular information on emerging pathogens mostly includes the sequence data, the methods that rely on sequences could be the most helpful approaches. This paper illustrates how sequence-oriented techniques and visualization approaches together can be drastically helpful for the interpretation of existing facts prior to the release of structural information obtained through more complicated and costly experiments. Many human pandemics have been rooted in host shifting. Introducing a fast and reliable approach to describe the emerging sequences will help us to tackle them and to discover effective medications and vaccinations.

## Methods

### Data sources

All sequences including the Sgp_SARS-CoV-2_ and its homologous sequences were obtained from the Uniprot Knowledge Base (UniprotKB)^79^ at www.uniprot.org (the accession number of Sgp_SARS-CoV-2_: P0DTC2; this reference sequence is one of the first sequenced Spgs of SARS-CoV-2).

The Immune Epitope Database (IEDB)^81^ was surveyed to extract the experimentally defined and validated linear and conformational epitopes of Sgp_SARS-CoV-2_.

### Hidden Markov model profiling

Similar sequences were collected by hidden Markov model profiling by HMMER software tool as provided by www.ebi.ac.uk. HMMER profiling simultaneously defines the domains on the protein sequence and collects homologous sequences from several optional databases. The database used for building the profile was the UniprotKB^82^. The data on the domains of Sgp_SARS-CoV-2_ were retrospectively collected from the available literature^82^ and automatic annotation of UniProtKB (www.uniprot.org). Following this procedure, major domains were defined and were separately searched against UniprotKB (www.uniprot.org) for collecting sequences similar to each domain.

The disparity index test^83^ was performed on all datasets to estimate the probability of rejecting the null hypothesis of substitution pattern heterogeneity. The judgment was stemmed from the extent of composition biases between the sequences. A Monte Carlo test with 500 replicates was employed for estimating the p-values^84^. The p-values lower than 0.05 were considered significant. The disparity index test was performed by the MEGAX software tool^83,85^ for each dataset separately.

### Clustering the sequence

Sequence clusters were built for all datasets by an all-against-all BLAST approach at MPI Bioinformatics toolkit by CLANS (CLuster ANalysis of Sequences) (https://toolkit.tuebingen.mpg.de/tools/clans)^84^, at a p-value of 10^–3^ and at least 1000 repulsions to avoid collapsing the nodes. The pairwise similarities were visualized in a graph by the CLANS stand-alone java application. The resulting CLANS files were further clustered by the network-based clustering function of the CLANS application. The network-based similarity clustering put similar sequences in separate groups, thereby making it easier to differentiate similar and dissimilar sequences in a complicated network of similarities.

Additionally, the overall mean distances in subpopulations and entire populations were estimated by the MEGAX software tool (ver. 10.1.7)^86,87^. The method allowed us to estimate the diversity of various groups. These groups were assigned in the datasets based on their viral genome origins.

### Alignments, analysis, and visualization

The MSAs were generated for all datasets by the Tcoffee algorithm^34^, as provided by the MPI bioinformatics toolkit at https://toolkit.tuebingen.mpg.de^88^. The alignments were then visualized and dissected by the Alvis alignment visualizer tool^89^. This alignment visualization as a sequence bundle by Alvis, has several useful features, including the precise definition of each position in the alignment, probing the harmonious location of certain amino acids in certain positions of any sequence in the MSA, and correspondence analysis, which are explained in the next section. The arrangement of letter-coded amino acids by physiochemical properties in the Y-axis of MSA vision makes the MSA presentations more informative. This physicochemical arrangement facilitates the sequence comparison and observation of the residual substitution effect(s).

### Correspondence analysis

The explorative interpretation of MSAs was done in a series of numerical experiments. The alignment kernels^90^ were computed for each MSA. The selected substitution matrix was BLASUM62. As numerical embedding, the Fisher scores of the emission probabilities^35^ were calculated by Alvis (ver. 0.1) after training a hidden Markov model^35^ on the MSAs. Then, the correspondence test^91^ was performed by Alvis (ver. 0.1). The correspondence test is an unsupervised (versus supervised) ordination method to detect dependencies between the sequences, sequence groups, and sites responsible for grouping in the alignment (for details see Ref.^92^).

### Structures

In addition to the characterization of homologous domains by HMMER, the sequence of Sgp_SARS-CoV-2_ was analyzed for locating the secondary structure elements and disordered regions. The sequence was analyzed by the RaptorX server (http://raptorx.uchicago.edu)^91^ and PSIpred (http://bioinf.cs.ucl.ac.uk/psipred)^93^ to reach a consensus position of the structural elements. SARS-CoV-2 related structures were obtained from the Protein Data Bank at www.rcsb.org, including 6VW1: 2019-nCoV chimeric receptor-binding domain complexed with its receptor, human ACE2, and 6VXX: Spg at its closed state^94^.

A homology modeling approach was also included to achieve a complete structure to avoid missing residues. The homotrimeric structure of Sgp_SARS-CoV-2_ was built by Galaxyhomomer^95^ at http://galaxy.seoklab.org/. The built structure was automatically refined based on the Cryo-electron microscopy structure of a coronavirus Spg trimer^96^ (PDB entry: 3JCL).

#### Network-based analyses

The molecular interactions of residues in protein structures (RBD and ACE2 complex; and furin cleavage site) were directly extracted from the tertiary structures by RINalyzer (ver. 2.0.0)^97^. The RINanalyzer enabled the connection of Cytoscape (ver. 3.7.2)^97^ with Chimera (ver. 1.13)^96,98^. The interaction networks were visualized and interpreted by Cytoscape (ver. 3.7.2)^99^. The hydrogen bonds, contacts, and distances (distance threshold < 5 Angstrom) were considered in the RINAnalyzer setting for extracting the network. Before network mining, the residues of interest were selected in Chimera (ver. 1.13). The network of interaction between the selected residues and neighboring residues was then extracted and visualized by Cytoscape (ver. 3.7.2).

### Centrality analysis

The key residues in the interaction networks were determined by the centrality analysis approach in the RINSpector software (ver. 1.1.0)^98^. The centrality measurement is based on the modification of the average shortest path length under the removal of individual residues^100^. This shortest path within two nodes (residues in the structure) is the path in the network that is required for connecting the first node to the second one with the minimum number of edges. This minimum number of edges is known as the shortest path length and the average shortest path length of all possible pairs of nodes identifies the average shortest path. The specific central residues were determined by calculating the Z-score; based on the alteration of an average shortest path length compared to the primary one. By increasing the average shortest path length upon removing a node, a Z-score would be increased. The Z-scores of greater than 2 were considered relevant^101^. The centrality analysis was done on the structures of RBD both as a free molecule and in complex with ACE2 (PDB entry: 6W41^101^). Similarly, centrality analysis was performed on the structure of the furin cleavage motif both in the free state and in complex with furin. The structure of the furin cleavage motif nestled in the furin active cleft was obtained by docking the predicted structure of the furin cleavage motif to unbounded furin (PDB ID: 5JXG^102,103^). The docking approach was based on the ZDOCK algorithm^104^ as provided by http://zdock.umassmed.edu.

### The evolutionary rate of RBM versus RBD

The physicochemical properties of the RBD sequence were based on amino acid scales of flexibility, surface accessibility, and buried area as calculated by the Protscale at www.expasy.ch^105^. The contact map of RBD was predicted using its sequence by RaptorX contact predict^100,106^ as provided by http://raptorx.uchicago.edu/. The contact map is indicative of interaction density; this interaction density here is inferred from the sequence data.

The identity and similarity scores to the RBD of Sgp_SARS-CoV-2_, from MSA, were mapped onto the structure of RBD (PDB entry: 6W41^106^; the structure was selected based on validation criteria). The mapping approach was based on the ProtSkin algorithm^106^ at http://www.mcgnmr.mcgill.ca/ProtSkin/. The conservation property of each site in RBD alignment was calculated even as the percentage of identity to the query sequence, or the average similarity score to the query sequence. The scores were calculated using the BLOSUM62 Block Substitution Matrix by the ProtSkin algorithm^100,107^. The obtained scores from the MSA file then were mapped onto the structure by a color gradient.

Protrusion (or convexity) index (CX), the depth of each atom in a protein structure (DPX), and the contact number of each residue were calculated by protein core/surface visualization workbench (PCW)^108^ at http://pongor.itk.ppke.hu/. These data along with B-factor were extracted from the PDB structure of RBD: 6W41^109^.

### Tracking the mutations in the Sgp_SARS-CoV-2_ amino acid sequences

To evaluate the positive or negative selections in RBD_SARS-CoV-2_ sequences, a collection of all available RBD_SARS-CoV-2_ sequences was built by a BLAST search against the available SARS-CoV-2 sequences. The best model with the lowest Bayesian Information Criterion scores was identified to describe the substitution pattern of this dataset. All positions containing gaps and missing data were completely deleted. The null hypothesis of d_N_ = d_S_ in favor of d_N_ > d_S_ or d_N_ < d_S_ was tested for tracing the positive or negative selections, respectively. These analyzes were performed in the MEGAX software (ver. 10.1.7).

Additionally, to monitor the mutation in each certain position (focal positions of this study), the mutation record of Sgp_SARS-CoV-2_ was obtained from the GISAID database^97,110^ at https://www.gisaid.org/. The replacement frequency of each position was examined to find any significant differences.

Distance difference for each pair of amino acids was also evaluated based on Grantham’s distances^97^.

### Statistical analysis

The nonparametric Mann–Whitney U test was performed to investigate the significant differences between certain positions and others. The selected positions were those that have been focused on in previous sections.

### Graphical visualization

Images were prepared by the CLANS Java application and graphical reporting tools of Chimera (1.13)^96,111^ and Cytoscape (3.7.2)^106^. The conservancy of amino acids of RBD was visualized by the PyMol graphic system (ver. 2.3.4)^106^ using the coloring macro generated by the ProtSkin^38,100^ based on similarity or identity scores.

## Supplementary Information

Supplementary Figure S1.

Supplementary Legends.

Dataset S1.

Dataset S2.

Dataset S3.

Dataset S4.

Dataset S5.

Supplementary Tables.

## Data Availability

All data associated with this study are present in the paper or the Supplementary Information.
