# Supplementary figures and images for: Hotspots for mutations in the SARS-CoV-2 spike glycoprotein: a correspondence analysis
Source: Sci Rep. 2021 Dec 8;11:23622. doi: 10.1038/s41598-021-01655-y (PMC8654821; doi:10.1038/s41598-021-01655-y)

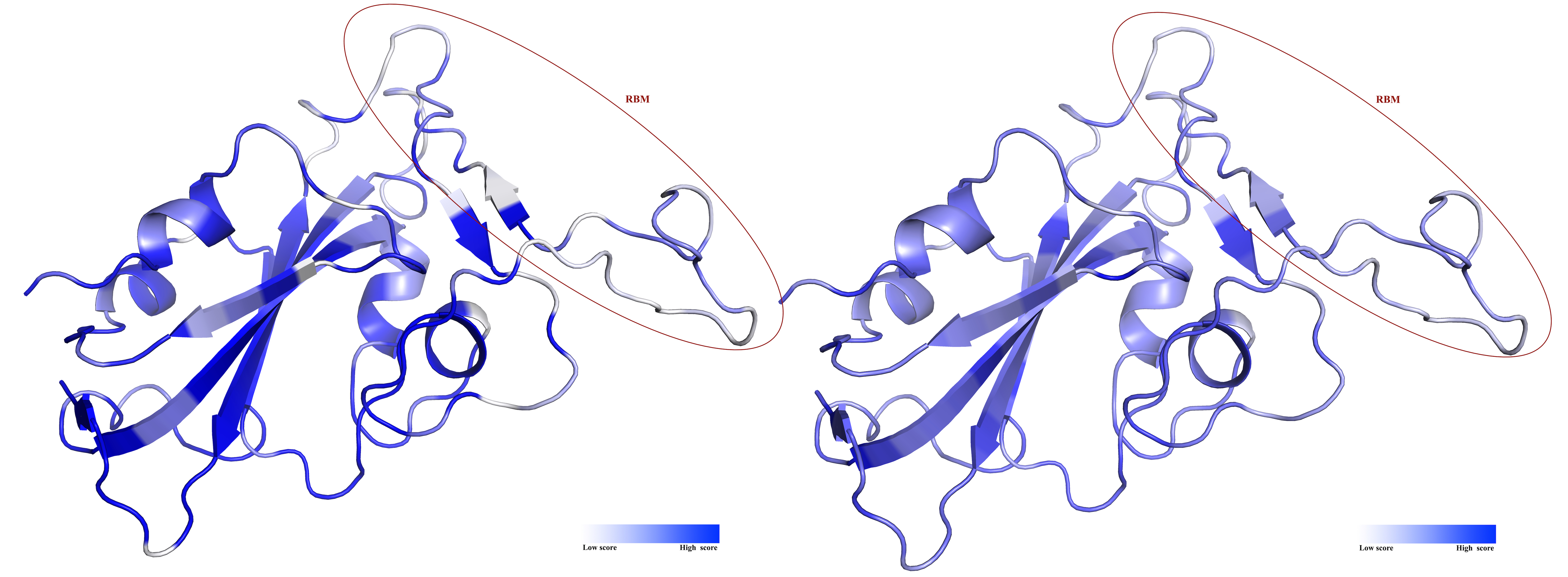

Supplement: Supplementary file 1 — Supplementary Figure S1. [file 41598_2021_1655_MOESM1_ESM.jpg]
