# Supplementary material for: Hotspots for mutations in the SARS-CoV-2 spike glycoprotein: a correspondence analysis
Source: Sci Rep. 2021 Dec 8;11:23622. doi: 10.1038/s41598-021-01655-y (PMC8654821; doi:10.1038/s41598-021-01655-y)
Supplement: Supplementary file 2 — Supplementary Legends. [file 41598_2021_1655_MOESM2_ESM.docx]

**Supplementary materials**

***Supplementary tables***

Table S1. Separation of spike glycoprotein of SARS-CoV-2.

Table S2. Residues involved in RBD and ACE2 interface; their Z-scores of centrality in complex and free form.

Table S3. The Grantham replacement scores for separated residues in this study.

Table S4. Residues involved in furin cleavage motif and furin interface; their Z-scores of centrality in complex and free form.

***Supplementary data***

***Supplementary data 1.*** Details of the cluster of sequences in CLANS analysis.

***Supplementary data 2.*** The list of sequence IDs used in the alignments.

***Supplementary data 3.*** Numerical scales of physicochemical properties of RBD residues.

***Supplementary data 4.*** Identity and similarity scores of RBD residues to homologous sequences in the alignment set.

***Supplementary data 5.*** All defined mutations in spike glycoprotein of SARS-CoV 2.

***Supplementary figure S1:*** The amino acid sequence of the spike glycoprotein of SARS-CoV-2. The major domains are annotated in different colors and labeled by a flag.
