# Supplementary material for: Hotspots for mutations in the SARS-CoV-2 spike glycoprotein: a correspondence analysis
Source: Sci Rep. 2021 Dec 8;11:23622. doi: 10.1038/s41598-021-01655-y (PMC8654821; doi:10.1038/s41598-021-01655-y)
Supplement: Supplementary file 8 — Supplementary Tables. [file 41598_2021_1655_MOESM8_ESM.docx]

Table S1. Domain definition of spike glycoprotein of SARS-CoV-2.

| **Domain Name** | **Position** | **InterPro** | **description** |
| --- | --- | --- | --- |
| **NTD** | 16-305 | IPR044341 | Betacoronavirus spike (S) glycoprotein S1 subunit N-terminal (NTD) domain |
| **RBD** | 330-521 | IPR018548 | This domain corresponds to the immunogenic receptor binding domain of the protein which binds to angiotensin-converting enzyme 2 (ACE2) |
| **Furin cleavage site** | 522-907 | - | Furin cleavage site containing part of spike glycoprotein |
| **HR1** | 908-985 | IPR002552 | Heptad repeat 1 |
| **CH** | 986-1035 |  | Central helix |
| **CD** | 1076-1141 |  | Connector domain |

Table S2. Residues involved in RBD and ACE2 interface.

| Residue | SS^*^ | Z-score in complex^**^ | Z-Score Free^**^ |
| --- | --- | --- | --- |
| ARG 408 | Helix | -0.456 | -0.462 |
| THR 415 | Loop | -0.456 | -0.908 |
| TYR 449 | Loop | -0.486 | -0.688 |
| TYR 453 | Sheet | -0.456 | 0.654 |
| LEU 455 | Loop | -0.456 | -0.643 |
| ALA 475 | Loop | -0.486 | 0.402 |
| PHE 486 | Loop | -0.353 | 0.554 |
| ASN 487 | Loop | -0.451 | 0.453 |
| TYR 489 | Loop | -0.451 | 0.942 |
| GLN 493 | Sheet | -0.392 | -0.64 |
| GLY 496 | Loop | -0.322 | -0.643 |
| GLN 498 | Loop | 0.192 | -0.761 |
| THR 500 | Loop | 1.332 | -0.303 |
| ASN 501 | Loop | 3.008 | -0.92 |
| GLY 502 | Loop | -0.394 | -0.627 |
| TYR 505 | Helix | 0.192 | -0.902 |

^*^ Secondary structure components

^**^ The centrality Z-scores of the interface residues of RBD in complex with ACE2 and free RBD

| **Amino acid in SARS-CoV-2 Sg** | **Amino Acids in the Respective Position** | **Grantham Replacement Score** |
| --- | --- | --- |
| **Thr 500** | Leu | 92 |
|  | Ala | 58 |
|  | Cys | 149 |
|  | Tyr | 92 |
|  | Ser | 58 |
|  | Asn | 65 |
| **Asn 501** | Lys | 94 |
|  | Ser | 46 |
|  | Thr | 65 |
|  | Ala | 111 |
|  | Ile | 149 |
|  | Leu | 153 |
|  | Val | 133 |
| **Gln 675** | His | 24 |
| **Gln 677** | Val | 96 |
|  | Ala | 91 |
| **Thr 678** | Ser | 58 |
| **Ala 684** | Ser | 99 |
|  | Gly | 60 |

Table S3. The Grantham replacement scores for separated residues in this study.

Table S4. Residues involved in furin cleavage motif and furin interface.

| Residue | SS | Z-score in Complex | Z-score Free |
| --- | --- | --- | --- |
| TYR 660 | Loop | 2.44 | 1.291 |
| CYS 662 | Loop | -0.726 | 1.99 |
| ILE 664 | Loop | -0.441 | -0.005 |
| GLY 669 | Loop | -0.899 | -0.442 |
| ILE 670 | Loop | -0.535 | 0.004 |
| CYS 671 | Loop | -0.139 | -0.265 |
| ALA 672 | Loop | -0.549 | -0.514 |
| SER 673 | Loop | -0.566 | -0.649 |
| TYR 674 | Loop | 1.828 | 0.549 |
| GLN 675 | Loop | -0.119 | -0.625 |
| THR 676 | Sheet | -0.102 | -0.337 |
| GLN 677 | Sheet | 2.772 | 0.556 |
| THR 678 | Sheet | 0.066 | 0.126 |
| ASN 679 | Sheet | -0.318 | 4.996 |
| SER 680 | Loop | 1.585 | 0.057 |
| PRO 681 | Loop | 0.43 | 0.116 |
| ARG 682 | Loop | 2.503 | 0.466 |
| ARG 683 | Helix | 0.62 | 2.738 |
| ALA 684 | Helix | -0.772 | 0.74 |
| ARG 685 | Helix | 0.509 | 1.577 |
| SER 686 | Helix | -0.466 | 0.923 |
| VAL 687 | Helix | -0.679 | -0.189 |
| ALA 688 | Helix | -0.663 | 0.231 |
| SER 689 | Helix | 0.348 | -0.051 |
| GLN 690 | Helix | -0.315 | 1.189 |
| SER 691 | Helix | -0.093 | -0.501 |
| ILE 692 | Loop | 4.29 | -0.046 |

^*^ Secondary structure components

^**^ The centrality Z- motif scores of interface residues of furin cleavage motif in complex with furin and free cleavage

Table S5: Experimentally validated linear epitopes of Sgp_SARS-CoV2._

| Link | Epitope type | Epitope Sequence/Residue | Start | End |
| --- | --- | --- | --- | --- |
| <http://www.iedb.org/epitope/1071575> | Linear peptide | NGVEGFNCYFPLQSY | 481 | 495 |
| <http://www.iedb.org/epitope/1075001> | Linear peptide | NGVEGFNCY | 481 | 489 |
| <http://www.iedb.org/epitope/1087656> | Linear peptide | NGVEGFNCYFPLQSYGFQ | 481 | 498 |
| <http://www.iedb.org/epitope/1071518> | Linear peptide | NCYFPLQSYGFQPTN | 487 | 501 |
| <http://www.iedb.org/epitope/1075121> | Linear peptide | YFPLQSYGF | 489 | 497 |
| <http://www.iedb.org/epitope/1087797> | Linear peptide | YFPLQSYGFQPTNGVGYQ | 489 | 506 |
| <http://www.iedb.org/epitope/1087346> | Linear peptide | FQPTNGVGY | 497 | 505 |
| <http://www.iedb.org/epitope/1087520> | Linear peptide | FQPTNGVGYQPYRVVVLS | 497 | 514 |
| <http://www.iedb.org/epitope/1310002> | Linear peptide | VVLSFELL | 497 | 504 |
| <http://www.iedb.org/epitope/1075016> | Linear peptide | PTNGVGYQPYRVVVLSFELLHAPATVCGPKKSTNLVKNKCVNF | 499 | 541 |
| <http://www.iedb.org/epitope/1069652> | Linear peptide | GAGICASYQTQTNSP | 667 | 681 |
| <http://www.iedb.org/epitope/1075070> | Linear peptide | SYQTQTNSPRRARSVA | 673 | 688 |
| <http://www.iedb.org/epitope/1087736> | Linear peptide | SYQTQTNSPRRARSVASQ | 673 | 690 |

Table S6: Experimentally validated conformational epitopes of Sgp_SARS-CoV2._

| Hyperlink | Epitope Type | Epitope residues |
| --- | --- | --- |
| <http://www.iedb.org/epitope/1087266> | Discontinuous peptide | R403, D405, R408, T415, G416, K417, D420, Y421, Y453, L455, F456, R457, K458, S459, N460, Y473, Q474, A475, G476, S477, F486, N487, Y489, Q493, S494, Y495, G496, Q498, T500, N501, G502, Y505 |
| <http://www.iedb.org/epitope/1087267> | Discontinuous peptide | R403, D405, T415, G416, K417, D420, Y421, Y453, L455, F456, R457, K458, N460, Y473, A475, G476, S477, F486, N487, Y489, Y495, N501, Y505 |
| <http://www.iedb.org/epitope/1087822> | Discontinuous peptide | T500, N501, G502 |
| <http://www.iedb.org/epitope/1310038> | Discontinuous peptide | R403, D405, T415, G416, K417, D420, Y421, Y453, L455, F456, R457, K458, N460, Y473, A475, G476, S477, F486, N487, Y489, Q493, S494, Y495, G496, T500, N501, G502, Y505 |
| <http://www.iedb.org/epitope/1311247> | Discontinuous peptide | R403, T415, G416, K417, D420, Y421, Y453, L455, F456, R457, K458, N460, Y473, A475, G476, S477, F486, N487, Y489, Q493, S494, Y495, G496, Q498, T500, N501, G502, V503, Y505 |
| <http://www.iedb.org/epitope/1311248> | Discontinuous peptide | R403, T415, G416, K417, D420, Y421, Y453, L455, R457, K458, S459, N460, Y473, Q474, A475, G476, S477, F486, N487, Y489, Q493, Q498, T500, N501, G502, V503, Y505 |
| <http://www.iedb.org/epitope/1311249> | Discontinuous peptide | R403, V445, G446, Y449, Y453, L455, F456, N487, Y489, Q493, Y495, G496, Q498, P499, T500, N501, G502, Y505 |
| <http://www.iedb.org/epitope/1311250> | Discontinuous peptide | T415, G416, K417, D420, Y421, Y453, L455, F456, R457, K458, S459, N460, Y473, Q474, A475, G476, S477, F486, N487, Y489, Q493, S494, Y495, G496, Q498, T500, N501, G502, Y505 |
